# Fremanezumab for Migraine Prevention in Japanese Elderly Aged Over 70 Years Old

**DOI:** 10.7759/cureus.34052

**Published:** 2023-01-22

**Authors:** Masahito Katsuki, Kenta Kashiwagi, Shin Kawamura, Senju Tachikawa, Akihito Koh

**Affiliations:** 1 Department of Neurosurgery, Itoigawa General Hospital, Itoigawa, JPN; 2 Department of Neurology, Itoigawa General Hospital, Itoigawa, JPN

**Keywords:** headache, migraine, elderly, calcitonin gene-related peptide-related monoclonal antibodies, adherance

## Abstract

Background

Elderly migraine is a public health problem with prolonged life expectancy, and effective prophylactic treatment is needed. There were no reports on fremanezumab for the elderly. We described the real-world data of fremanezumab, calcitonin gene-related peptide-related monoclonal antibody (CGRP-mAB), for migraine in elderly over 70 years old.

Methods

We retrospectively investigated six elderly migraine patients over 70 years old treated with fremanezumab. Headache impact test-6 (HIT-6), monthly headache days (MHD), and monthly acute medication intake days (AMD) before one and three months after starting fremanezumab treatment were evaluated.

Results

Three women and three men (median age 78; range: 71-99) were included. One was chronic migraine (CM), three were CM and medication-overuse headache, and two were episodic migraine and tension-type headache. All six patients received monthly fremanezumab. The median MHD before, during one, and three months after treatment were 30 (4-30), 30 (4-30), and 29 (15-30, n=4). Those of AMD were 17 (0-30), 9.5 (0-30), and 1 (0-28). Those about HIT-6 were 64 (56-72), 59.5 (52-70), and 55.5 (48-64). Two (33.3%) of the six patients experienced therapeutic effectiveness. There were no side effects.

Conclusion

We described the six elderly migraine patients aged over 70 years old treated with fremanezumab. Two (33.3%) of the six patients experienced therapeutic effectiveness. This is the first report of fremanezumab for elderly migraine patients aged over 70 years old. Further evidence accumulation is needed about CGRP-mABs for the elderly.

## Introduction

Migraine is a widespread public health problem [1]. In Japan, migraine prevalence is 4.3-8.4% [2-5], and 29.8-74.2% of patients with migraine headaches report that it significantly impairs their daily activity [1]. Also, the migraine prevalence among elderly aged over 64 years old is 0.9%, and those of medication-overuse headache (MOH) is 0.7% in Japan [6]. Effective prophylactic treatment for the elderly with migraine is needed. However, the classical prophylactic treatment has limited efficacy and is sometimes associated with side effects that can result in poor adherence [7,8]. Furthermore, prophylaxis of the elderly’s migraine may be more challenging due to multiple comorbidities and polypharmacy [9]. In this context, effective prophylactic medication for migraine with good adherence and fewer side effects is needed. Fremanezumab, a calcitonin gene-related peptide-related monoclonal antibody (CGRP-mAB), can be used for migraine prevention in adult patients aged 18 years or older [10]. The pooled analysis of the three randomized placebo-controlled trials, the HALO EM study (ClinicalTrials.gov identifier: #NCT02629861: Efficacy and safety of 2 dose regimens of fremanezumab versus placebo for the preventive treatment of episodic migraine), HALO CM study (#NCT02621931: Comparing efficacy and safety of 2 dose regimens of subcutaneous administration of fremanezumab versus placebo for the preventive treatment of chronic migraine), and FOCUS (#NCT03308968: An efficacy and safety study of fremanezumab in adults with migraine), revealed that fremanezumab treatment is efficacious and well-tolerated over 12 weeks in episodic migraine (EM) and chronic migraine (CM) in patients aged 60-70 years old [11]. The changes in monthly migraine days from baseline over 12 weeks were -4.3 days for quarterly fremanezumab of 675 mg and -4.6 days for monthly fremanezumab of 225 mg versus -2.3 days for placebo. They have a growing interest in their application in elderly’s migraine aged over 70 years old, but there were no such reports. We herein presented six elderly migraine patients aged over 70 years old treated with fremanezumab as an early clinical experience.

## Materials and methods

Study population

We investigated our hospital’s electronic medical records between September 2021 and December 2022 and evaluated six elderly migraine patients aged over 70 years old treated with fremanezumab at our headache-specialized outpatient. The patients suffered headaches for at least 90 days before the fremanezumab treatment and had been keeping headache diaries. They had had at least one type of prophylactic medication before fremanezumab use, but it failed. The headache diagnosis was based on the International Classifications of Headache Disorder 3rd edition (ICHD-3) [12]. CM, chronic tension-type headache (CTTH), EM, tension-type headache (TTH), and MOH were diagnosed. We acquired written informed consent from all the patients or patients’ families for this study. We performed this retrospective study according to the Declaration of Helsinki.

Clinical variables and outcomes

We collected patients’ characteristics, such as age, sex, and modified Rankin Scale [13], indicating activities of daily living, medical history, medication for comorbidities, and previous prophylactic medication. Clinical data reported by paper-based or electronic headache diaries were used. Monthly headache days (MHD) and acute medication intake days (AMD) were defined as the monthly values over the respective observation period of 30 days. Head impact test-6 (HIT-6) [14] was also investigated over the respective observation period. The outcomes were defined as the changes in MHD, AMD, and HIT-6 before treatment, after one and three months. We defined a three-month HIT-6 score under 50 as therapeutically effective. Results were presented as median (range).

## Results

Table 1 shows the characteristics of six elderly migraine patients aged over 70 years old treated with fremanezumab.

**Table 1:** Patient characteristics. AMD: Monthly acute medication intake days; CM: Chronic migraine; CTTH: Chronic tension-type headache; EM: Episodic migraine; HIT-6: Headache Impact Test-6; MHD: Monthly headache days; MOH: Medication-overuse headache; mRS: modified Rankin Scale; TTH: Tension-type headache.

No.	Age	Sex	Diagnosis	Modified Rankin Scale	Medical history	Medication	Failed prophylaxis	MHD	AMD	HIT-6	1-month MHD	1-month AMD	1-month HIT-6	3-month MHD	3-month AMD	3-month HIT-6	Follow-up
1	71	M	CM+MOH	mRS 0 (working, driving)	Bronchial asthma	Theophylline, vilanterol	Valproic acid, goreisan	30	30	56	30	15	49	30	0	48	3month, continued
2	72	W	EM+TTH	mRS 0 (working, driving)	Constipation, anxiety, suicidal ideation	Magnesium oxide	Propranolol	30	2	72	15	2	52	15	2	49	3 month, continued
3	76	W	CM+MOH	mRS 0 (neither working, nor driving)	Hypertension, depression, suicidal ideation	Zolpidem, olanzapine, azilsartan, risperidone, levomepromazine	Valproic acid	4	4	62	4	4	57	28	28	64	3 month, swtiched to erenumab
4	80	M	CM+MOH	mRS 0 (not working, but driving)	Hypertension, cerebral infarction	Aspirin, olmesartan, nifedipin	Valproic acid, amitriptyline	30	0	63	30	0	62	30	0	62	3 month, stopped due to ineffectiveness
5	90	M	CM	mRS 2 (stay home, living with family)	Hypertension, bladder carcinoma, cerebral infarction, reflux esophagitis	Theophylline, pranlukast, carbocysteine, indacaterol, omeprazole, azilsartan, amlodipine, aspirin, azosemide	Olmesartan, yokukansan	30	30	72	30	30	70	-	-	-	1 month, admitted to other hospital due to urinary tract infection
6	99	F	EM+CTTH	mRS 3 (stay home, living with family)	Hypertension, lower back pain, postoperative femur fracture, mild cognitive impairment	Pregabalin, benidipine, clonazepam, sulpiride	Goreisan, yokukansan	30	30	65	30	30	64	-	-	-	1 month, stopped due to ineffectiveness

**Figure 1:**
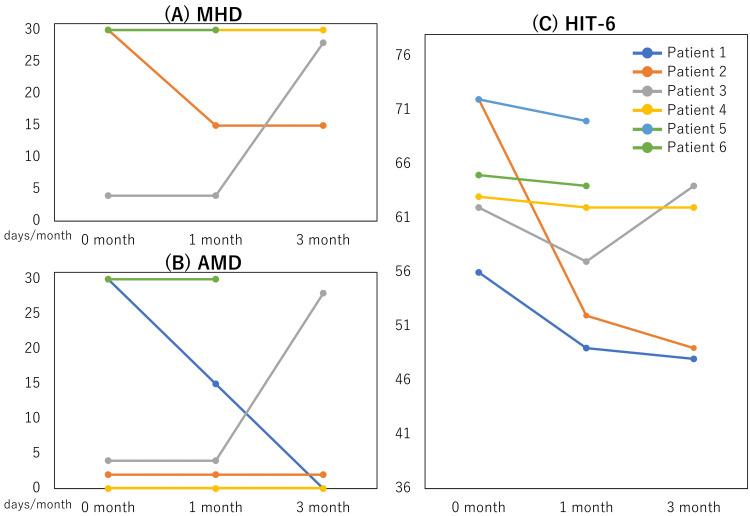
Therapeutic effects. Chronological changes of monthly headache days (MHD) (A), monthly acute medication intake days (AMD) (B), and Headache Impact Test-6 (HIT-6) scores (C) after starting fremanezumab treatment (0 months).

Three women and three men were included. The median age was 78 (71-99). Of the six patients, one was CM, three were CM+MOH, one was EM+TTH, and one was EM+CTTH. All six patients received monthly fremanezumab of 225 mg. Three of the six took Japanese herbal Kampo medicine [15,16] as prophylaxis before fremanezumab treatment. Patients 1 had CM and MOH, and took valproic acid and goreisan. Fremanezumab rapidly had efficacy decreasing AMD and HIT-6. However, MHD did not decrease. Patient 2 had EM and TTH. MHD, AMD, and HIT-6 decreased during the treatment period. MHD was 15 days, but the headache seemed TTH-like. Patients 1 and 2 were satisfied with fremanezumab and continued. Fremanezumab was ineffective for patient 3, so we switched to erenumab, another CGRP-mAB. After switching, erenumab seemed relatively effective compared to fremanezumab, and we had followed up on the efficacy. For patient 4, fremanezumab was not effective after three months, so she stopped. We are now trying to use other oral prophylaxis like Kampo medicine. Patient 5 did not take the second fremanezumab dose because he was admitted to another hospital due to a UTI, so we could not follow up. Patient 6 did not take the second fremanezumab due to the ineffectiveness and stopped consulting us. Therapeutic effects are described in Figure 1.

The median MHD before, one, and three months after treatment were 30 (4-30), 30 (4-30), and 29 (15-30, n=4) (Figure 1A). AMD before, one, and three months after treatment were 17 (0-30), 9.5 (0-30), and 1 (0-28) (Figure 1B). Those about HIT-6 were 64 (56-72), 59.5 (52-70), and 55.5 (48-64) (Figure 1C). Two (33.3%) of the six patients (patients 1 and 2) experienced therapeutic effectiveness. There were no side effects of fremanezumab.

## Discussion

We herein described a case of six elderly migraine patients aged over 70 years old treated with fremanezumab. Two (33.3%) of the six patients (patients 1 and 2) experienced therapeutic effectiveness. There were no side effects of fremanezumab. This is the first experience of fremanezumab for elderly migraine patients aged over 70 years old. The advent of CGRP-mABs has provided a new treatment option for the elderly with migraine. However, the clinical trials that evaluated the security and effectiveness of CGRP-mABs did not include the elderly (adults older than 65 years old were excluded from the galcanezumab and erenumab CM and EM studies; adults older than 70 years old were excluded from the fremanezumab CM and EM studies) [11]. The effectiveness and safety of these medications in the senior population have not been thoroughly studied in real-world settings. Patel P et al. presented the efficacy of CGRP-mABs for 32 elderly patients (28 women and four men) aged over 65 years old. In the report, 30 were treated with erenumab, one with fremanezumab, and one with galcanezumab. The age ranged from 65 to 84 years. Of the 32 elderly, 17 (53.1%) experienced a reduction in headache frequency, 14 (43.8%) felt no change, and one (3.1%) had an increase in headache frequency. On average, headache frequency was reduced by 13.9 days (-39.7%). Constipation and nausea were the most prevalent side effects, which were reported by five patients (15.6%) [17]. Combined with Patel P et al. report, CGRP-mABs can have efficacy for some of the elderly's migraine. However, the heterogeneity of the patient's characteristics and severity of headaches differ from study to study, so consistent effectiveness was not demonstrated of the CGRP-mABs for the elderly. In our cases, only two of six patients experienced the fremanezumab's efficacy. The older age of the patients may suffer from the longer duration of migraine, together with CM and MOH, leading to changes in the brain's structure [18,19] and making it more difficult for the CGRP-mABs to be effective. In such cases, erenumab, which has been suggested to have the potential to improve the functional structure of the brain [20], maybe a better choice. It is also possible that those who were ineffective were already taking several medications, and they may have modified the CGRP-mABs effects.

As people get older, headache conditions and migraines become less common [2], although healthy life expectancy becomes longer. According to a Japanese survey, the prevalence of the elderly's headache disorders, migraines, and MOH was 11.97%, 0.91%, and 0.70%, respectively [6]. However, the true prevalence of migraine in the elderly is potentially high compared to these results. This is because the clinical characteristics of migraine change with age and as comorbidities increase, meaning that migraine in the elderly is becoming less migraine-like, and we may miss migraine in the elderly. As men get older, their prevalence of migraines significantly declines. As women get older, the duration of headaches, unilateral pain, pulsating sensation, light sensitivity, and noise sensitivity increases. Aging in women reduces the tendency of headaches to get worse with exercise. Elderly people's neck pain also worsens with severe migraine attacks [21]. Considering the headache characteristics change related to age, the expansion of indications for CGRP-mABs may be suggested. The first time we see an elderly patient at a headache clinic, his or her migraine may have morphed into a headache as if it were not a migraine. It may be necessary to ask about the patient's history of migraine when he or she was younger. Suppose there is a history of migraine in the past. In that case, even if the headache at the current visit does not necessarily correspond to ICHD-3-defined migraine, it may be considered a transformation of migraine in the past, and the indication of CGRP-mABs may be indicated. Further evidence accumulation is needed about CGRP-mABs for the elderly. We herein described the limitation. The present results were based on a small number of cases at a single institution in Japan. Therefore, it remains unknown whether the results will be similar for elderly migraine patients overseas. Fremanezumab injections may have triggered the patients to keep a headache diary and to think about their health more carefully. It is also possible that other prophylactic drugs may have had a delayed effect. Further investigation with a large population is needed.

## Conclusions

We herein described a case of six elderly migraine patients aged over 70 years old treated with fremanezumab. Two (33.3%) of the six patients (patients 1 and 2) experienced therapeutic effectiveness. There were no side effects of fremanezumab. This is the first report of fremanezumab for elderly migraine patients aged over 70 years old. Some older adults also have their lives interfered with by migraine, and the efficacies of CGRP-mABs on the elderly is expected. Further evidence accumulation is needed about CGRP-mABs for the elderly.
